# Ozone-Microbubble-Washing with Domestic Equipment: Effects on the Microstructure, and Lipid and Protein Oxidation of Muscle Foods

**DOI:** 10.3390/foods11070903

**Published:** 2022-03-22

**Authors:** Mei-Hui Pian, Lu Dong, Zhen-Ting Yu, Fan Wei, Chun-Yang Li, Dan-Cai Fan, Shi-Jie Li, Yan Zhang, Shuo Wang

**Affiliations:** Tianjin Key Laboratory of Food Science and Health, School of Medicine, Nankai University, Tianjin 300350, China; mhpian@mail.nankai.edu.cn (M.-H.P.); donglu@nankai.edu.cn (L.D.); 2120191293@mail.nankai.edu.cn (Z.-T.Y.); weifan@mail.nankai.edu.cn (F.W.); sunnee@163.com (C.-Y.L.); fandancai@163.com (D.-C.F.); lsj930427@126.com (S.-J.L.); yzhang@nankai.edu.cn (Y.Z.)

**Keywords:** ozone-microbubbles-washing, ozone, domestic ozone equipment, washing treatment, pork, fish, oxidation

## Abstract

This study aimed to compare ozone-microbubble-washing (OM) performed by domestic equipment with conventional water-washing (CW) regarding resultant quality attributes of muscle foods. For this purpose, muscle microstructure and lipid and protein oxidation were evaluated in pork and fish samples after OM and CW treatments. The assessment of muscle microstructure showed that OM treatment did not damage the microstructure of muscle fibers in both pork and fish samples. Thiobarbituric acid reactive substances (TBARS) values were not detected in both treatment groups, and they were substantially below the generally acceptable threshold (1 mg MDA/kg). The methylglyoxal (MGO) level of OM-treated fish samples was significantly higher than that of CW-treated fish samples. However, glyoxal (GO) and MGO levels of OM-treated pork samples were significantly lower than that of CW-treated pork samples. Similar types and sites of oxidative modification and similar numbers of modified peptides, as well as no significant difference in the concentration of total and most of the free amino acids (FAA) between treatment groups, indicated that OM treatment did not accelerate protein oxidation.

## 1. Introduction

Ozone is a strong oxidant only second to fluorides [1], which has strong oxidative effects and can kill various pathogenic microorganisms such as viruses, bacteria, and fungi, without producing toxic residues [2]. Hence, it has been considered as a green alternative to chlorine compound disinfectants and has been widely applied in the environmental, medical, and food industries [1,3,4]. Since it was classified by the US Food and Drug Administration as a “Generally Recognized As Safe” process and food additive, it has been widely used in the food industry as a non-thermal processing technology [5].

Ozone has been widely applied as a sanitizer to improve the microbial safety of foods. Horvitz et al. [6] found that Andean blackberries treated with 0.7 ppm gaseous ozone for 3 min during 10 days of cold storage (6 ± 1 °C) have the best microbial quality (with the lowest microbial populations) without physiological damage compared with those untreated and treated with three lower doses of gaseous ozone (0.4, 0.5, and 0.6 ppm). Zhao et al. [7] evaluated the effects of ozone-water-washing (4.5 mg/L, 30 min) on the quality of Nile tilapia fillets for 180 days (−18 ± 1 °C), and the results showed that the textural quality was enhanced and shelf life prolonged compared with the untreated fillets. Furthermore, ozone has proved to be effective in increasing the lightness value of foods [8], removing odor [9], and sanitizing equipment in food plants [10]. In addition, the ability of ozone to remove pesticide residues in foods has intensively been studied. For instance, 1.4 mg/L ozone-water-washing for 30 min was considered as an effective way to remove six pesticide residues and improve shelf life and safety of fresh-cut cabbage [11].

Based on the advantages of ozone treatment in improving microbial safety, degrading pesticides, improving food quality, and extending food shelf life, an increasing number of small domestic ozone equipment has been developed and applied in kitchen cleaning. Previous studies reported that the aerobic plate count value and total viable count reduction ratio of shrimp treated by ozone-washing with domestic ozone equipment was significantly lower during the iced storage of days compared with control (untreated ice-stored shrimp), which indicates that the ozone-washing with domestic ozone equipment is effective against microorganisms as a sanitizer [12,13]. Furthermore, the combination of ozone with microbubble technology used by some novel equipment has been proven to be an effective cleaning treatment in reducing microbial populations and removing contaminants such as pesticide residues [14,15,16]. The primary mechanism of ozone as a disinfectant is to induce strong oxidation. Nevertheless, there are few reports on whether or not ozone-microbubble-washing with the domestic ozone equipment will cause immediate oxidation of nutrients such as fat and protein in foods.

Thus, more specific assessments of food qualities using domestic equipment for ozone-water-washing should be considered. However, to our best knowledge, only some studies have evaluated proximate composition (fat, moisture, and protein contents), color and textural attributes (adhesiveness, fracturability, and hardness) [17], and lipid oxidation kinetics of ozone-treated shrimp during cold or iced storage [18]. On the one hand, the previous investigations mainly focus on the impact of ozone-washing by domestic equipment on food quality after a long-term cold or iced storage. Given the practical conditions in conventional households, washed foods are usually eaten immediately. Therefore, more attention should be paid to the immediate impact of ozone-washing with domestic ozone equipment on the quality of foods compared with conventional household washing. On the other hand, although the lipid oxidation of shrimp was evaluated, few studies assessed other attributes, such as protein oxidation, of other foods. Therefore, to supplement the existing knowledge on the immediate impact of ozone-microbubble-washing with domestic ozone equipment on the quality of foods compared with conventional household washing, the specific objective of this study was to assess the microstructure and lipid and protein oxidation of selected muscle foods, namely pork and fish, which is of practical significance.

## 2. Materials and Methods

### 2.1. Samples

Fresh pork tenderloin (*Psoas major*) and grass carp (*Ctenopharyngodon idella*) were purchased from the local retail market (Tianjin, China) and were transported in flake ice (1:1) to the laboratory within 20 min.

### 2.2. Treatments

Each species of muscle foods including pork tenderloin (pork) and grass carp (fish), was subdivided into two groups: food treated with conventional water-washing (CW); food treated with ozone-microbubble-washing (OM). The weights of treated pork (in an approximately 15.5 cm × 5.5 cm × 6.0 cm cuboid shape as a whole) and eviscerated fish were 0.5 kg and 1.0 kg, respectively, for each treatment group.

OM treatment was carried out by the US-I05 ozone-microbubble equipment (Zhejiang Uish Environmental Technology Co., Ningbo, China) with five functions. The working principle of this equipment is that ozone was generated by corona discharge combined with the microbubbles to increase the cleaning effectiveness. For OM, the washing times of pork and fish were 12 min (±0.1 min) and 15 min (±0.1 min), respectively, according to the equipment setting instructions. The concentration of aqueous ozone generated during washing was 1.57 mg/L, which was determined by the method according to GB/T 5750.11-2006. During the two treatments, the temperature was maintained at room temperature (25 ± 2 °C), and the temperature of water in each treatment was 20 ± 1 °C. As contrasted with OM, CW was used to closely simulate conventional household washing treatment (washed with equivalent water for the same time).

The muscles of pork samples from the surface, and fish samples from the right-back surface were cut into 1.0 cm × 1.0 cm × 0.5 cm cubes (*n* = 3) immediately after OM or CW treatment and fixed in 4% formalin for the assessment of muscle microstructure. Then, the head, tail, skin, and bone of fish were removed. The muscles of all samples were ground separately for 30 s in food processors until homogeneous for chemical analysis.

### 2.3. Assessment of Muscle Microstructure

The histomorphology of muscle was analyzed by the method of Song et al. [19] with minor modifications. The samples fixed in 4% formalin for 24 h were then cut into 5 mm × 5 mm × 5 mm blocks, embedded in paraffin, and cut into 4 μm thick sections before being stained with hematoxylin-eosin stain kit (G1120, Solarbio, Beijing, China). The tissue was observed by micro 3DHISTECH P250 FLASH (3DHISTECH, Budapest, Hungary), and images were captured using CaseViewer V2.3 (3DHISTECH, Budapest, Hungary) software with a 10× magnification.

### 2.4. Quantification of Thiobarbituric Acid Reactive Substances (TBARS) Values

The lipid peroxidation was determined using the TBARS method described by the standard of GB/T 5009.6-2003 (Chinese National Standards: determination of malondialdehyde in food), and the results were reported as milligrams malondialdehyde (MDA)/kilogram.

### 2.5. Quantification of Glyoxal (GO) and Methylglyoxal (MGO)

#### 2.5.1. Sample Pretreatment

GO and MGO were extracted and derivatized following the procedure reported by Zhang et al. [20] with some modifications. Briefly, 5 g of sample was vortexed with 10 mL of water for 3 min, and then the mixture was centrifuged at 10,000 r/min for 10 min. A second extraction was applied under the same conditions as above. The supernatant of twice extraction was collected, and 10 mL of *n*-hexane (Aladdin, Shanghai, China) and 0.5 mL of Carrez reagents I and II (Aladdin, Shanghai, China) were added. After vortexing for 5 min followed by centrifugation for 10 min at 10,000 r/min, 3 mL of the middle layer of supernatant was transferred into a brown centrifuge tube. Then, 100 μL of *O*-phenylene-diamine (20 mg/mL) was added to the tube and shaken at 100 r/min for 20 min (60 °C). Subsequently, the methanol was loaded through an Oasis^®^ HLB cartridge column with a capacity of 6 cc (200 mg, Waters, Milford, MA, USA). The column was activated using 5 mL of methanol, followed by equilibration with 5 mL ultrapure water. Then, the derivative sample solution was loaded through it. The cartridge was subsequently cleaned with 5 mL ultrapure water, and the analytes were eluted by 3 mL of acetonitrile/water (1/1, *v*/*v*). An amount of 30 μL of *O*-phenylene-diamine (20 mg/mL) was mixed with 1 mL diluted standard solution (Sigma-Aldrich, St. Louis, MO, USA), followed by derivatization in the same condition. All solutions were filtered through 0.22 μm filters before being injected into UPLC-MS/MS.

#### 2.5.2. UPLC-MS/MS Analysis

GO and MGO were separated and analyzed by Waters Acquity UPLC I-class coupled to a Waters Xevo TQ-S Micro triple tandem quadrupole mass spectrometer (Waters, Milford, MA, USA). Chromatographic separations were carried out on Waters Symmetry C18 column (4.6 mm × 150 mm, 5 μm) which was eluted with methanol (phase A) and 0.1% (*v*/*v*) formic acid–water (phase B) at a flow rate of 0.5 mL/min. The gradient elution program was 0–10 min, 70–40% B; 10–12 min, 40–70% B; 12–15 min, 70% B. The MS condition was set according to the method reported by Wang et al. [21].

### 2.6. Quantification of Free Amino Acids

#### 2.6.1. Sample Pretreatment

An amount of 3 g of sample was ultrasonicated with 20 mL of 0.2 mol/L phosphate buffer solution for 20 min. The mixture was stored at 4 °C for 18 h after vortexing with 20 mL of 5% trichloroacetic acid (TCA) for 1 min. Next, the mixture was centrifuged for 20 min, the supernatant was collected after being filtered through Whatman No. 4 filter paper (Whatman International Co., Ltd., Maidstone, UK), followed by being adjusted to pH 6.0–6.2 with 4 mol/L NaOH. Afterward, the filtrates were transferred into a volumetric flask and diluted to 50 mL with ultrapure water, followed by filtration through a 0.22 μm membrane. Then, 10 μL of sample filtrate and the amino acid standard solution were derivatized as reported by Goh et al. [22].

#### 2.6.2. HPLC Analysis

The separation was performed on a Nova-Pak C18 Column (150 mm × 3.9 mm, 4 μm; Waters, Milford, MA, USA) at 37 °C. The injection volume was 10 μL. The chromatographic conditions were chosen according to a previously reported method [23], using an Alliance 2695 HPLC system with a 2475 Multi λ Fluorescence detector (Waters, Milford, MA, USA). The concentration of amino acids from samples was identified and quantified by comparison with the amino acid mixture (AAS18; Sigma-Aldrich, St. Louis, MO, USA).

### 2.7. Identification of Oxidation Sites on Proteins

#### 2.7.1. Protein Extraction

An amount of 200 mg of the muscle emulsion sample was homogenized in the buffer solution (2% SDS, 0.1 mol/L KCl, 0.02% sodium azide, 20 mmol/L Tris-HCl, pH 7.5) for 30 s and incubated at 37 °C for 10 min. Next, a second homogenization was performed under the same conditions as above after the samples cooled down to room temperature. The mixture was centrifuged for 10 min, and the supernatant was then collected, with protein concentration quantified by the BCA method.

#### 2.7.2. Protein Digestion

An amount of 200 μg of proteins of each sample was reduced according to the literature [24]. Then, iodoacetamide was added to a final concentration of 50 mmol/L, followed by incubation for 40 min at room temperature in the dark. Subsequently, the solution was filtered by a 10 kDa unit (Millipore Co., Ltd., Bedford, MA, USA) and centrifuged at 12,000× *g* for 30 min. The filter was washed twice with 400 μL of 8 mol/L urea solution and was washed three times with 400 μL of 50 mmol/L NH_4_HCO_3_ solution. Thereafter, the protein precipitates were digested in 200 μL of 50 mmol/L NH_4_HCO_3_ solution and subjected to digestion at 37 °C for 16 h by trypsin (enzyme-to-substrate ratio 1:50, *w*/*w*), followed by centrifugation at 12,000× *g*. The supernatants were collected and freeze-dried to collect the peptides.

#### 2.7.3. High-Resolution Mass Spectrometry (HR-MS) Analysis

Easy Nano LC1200 UPLC system coupled to an Orbitrap Fusion Lumos Mass Spectrometer (Thermo Scientific, San Jose, CA, USA) was used to detect the oxidation of proteins. The dried peptides were dissolved in 0.1% formic acid solution and loaded onto a homemade C18 column (75 μm × 150 mm, 3 μm) eluted by mobile phase according to the previous literature [25] at the flow rate of 300 nL/min with gradient (5–10% solvent B for 4 min, followed by 10–28% over 36 min, 28–38% over 7 min, 38–100% over 1 min, and 100% over 12 min). The MS condition set as previously described [25] with minor modifications was as follows: ion spray voltage 2.1 kV, parent ion scan range *m*/*z* 350–1550, daughter ion scan range from *m*/*z* 110, maximum ion injection time 50 ms, dynamic exclusion 60 s. Peptides were searched in the UniProtKB database (Sus scrofa and Xenocypridinae) according to the literature [26]. Table 1 presents the list of identified oxidative modifications [26]. In this work, −10lgP (probability of false discovery) score ≥ 15 was used to filter out non-confident identifications.

### 2.8. Statistical Analysis

Three independent repetitions of the experiment were performed, and the experimental results were subjected to an independent sample *t*-test by SPSS 25.0 software (SPSS Inc., Chicago, IL, USA) and were considered significant at *p* < 0.05.

## 3. Results and Discussion

### 3.1. Effects of OM Treatment on Microstructure of Muscle Tissue

**Figure 1:**
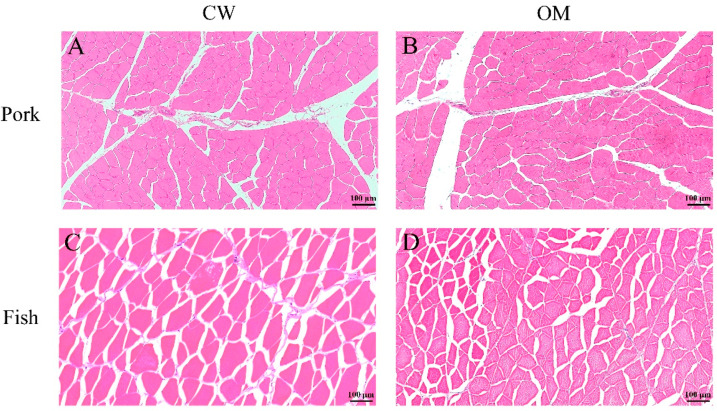
Effects of different washing treatments on the microstructure of muscle tissue. CW: conventional water-washing; OM: ozone-microbubble-washing. Pork samples treated with CM (**A**) and OM (**B**); fish treated with CW (**C**) and OM (**D**).

Since the damage of muscle fiber is correlated to the lipid and protein oxidation [27], as well as quality attributes, including the water-holding capacity and texture [28], histology was used to evaluate the effects of OM washing treatments on the microstructure of muscle tissue. The cross-sections of pork and fish tissues subjected to different washing treatments are shown in Figure 1. The cell nucleus appears blue, muscle fibers red, and the space between the muscle fibers white.

For the CW group of the pork sample (Figure 1A), the muscle fibers looked tightened and round, and were undulated and arranged freely relative to each other. Intact myofibrillar bundles and cellular structure, as well as clear boundaries, were found. For the OM group of pork samples (Figure 1B), the border between fibers appeared clear. The pork samples treated with OM exhibited the same microstructure of muscle fibers as the CW group of pork samples, with round and closely arranged fibers and clearly marked boundaries. Thicker perimysium and thinner endomysium were observed in both treatment groups of the pork samples. Overall, the pork samples treated with CW and OM treatments exhibited complete cell shapes, intact myofibrillar bundles, and similar extracellular spaces (Figure 1A,B).

For the CW group of the fish samples (Figure 1C), the muscle fibers were regularly arranged with great extracellular spaces, while the spacing between the muscle fibers from the OM group of fish samples (Figure 1D) was wide or narrow at random. Although the distance between muscle fibers was visible in both treatment groups, muscle fibers were polygonal in shape, and sarcolemma remained intact.

The microstructure observations allow us to conclude that OM treatment did not rupture the fiber membranes or damage the muscle cells, compared with CW treatment, which indicated OM treatment might not lead to the oxidation of cell contents.

### 3.2. Effects of OM Treatment on Lipid Oxidation

Lipid oxidation caused by reactive oxygen species (ROS) deteriorates the color, flavor, and tenderness of muscle foods [29]. The TBARS values and the content of GO and MGO were determined to evaluate the lipid oxidation.

#### 3.2.1. TBARS Values

TBARS is generally used as a marker of lipid peroxidation [30]. However, it was not detected in pork and fish samples treated with CW or OM (Table 2). Undetected TBARS values of samples may be due to the freshness of pork and fish samples and the limit of determination (LOD) of the colorimetric measure method. Since the LOD is 0.05 mg MDA/kg, indicating that the TBARS values of both pork and fish samples subjected by OM treatment were under 0.05 mg MDA/kg, the OM-treated samples were considered acceptable (TBARS < 1 mg MDA/kg) for consumption [31]. Several authors have also found that ozone treatment did not cause unacceptable TBARS values (1 mg MDA/kg). Zhao et al. [7] and Giménez et al. [32] reported that the TBARS values of muscle foods samples exposed by ozone treatment at day 0 were under the threshold (1 mg MDA/kg) and did not show a significant difference compared with that of untreated samples. Therefore, it is suggested that OM treatment in pork and fish samples was not harmful to TBARS values.

#### 3.2.2. GO and MGO

Since the colorimetric measurement of TBARS was not sensitive, to further investigate whether OM treatment promoted lipid oxidation, GO and MGO, the most toxic secondary products of the lipid oxidation [33], were selected and quantified using the UPLC-MS/MS method.

In the fish samples, there was no significant difference in the level of GO between OM treatment and CW treatment, but MGO content in the OM group was significantly (*p* < 0.05) higher than in the CW group (Table 2), indicating that OM treatment promoted the oxidation of lipid in fish, which is consistent with the pro-oxidation property of ozone [34]. However, in the pork samples, the levels of GO and MGO in the OM group were significantly (*p* < 0.05) lower than in the CW group (Table 2). GO and MGO are water-soluble compounds, and ozone-microbubble treatment may cause more GO and MGO dissolving in water, leading to a decrease in the residual GO and MGO content in foods within a certain concentration range. More MGO and GO were produced in fish than in pork samples under the same treatment conditions, which may be due to the higher fat content in fish samples. Therefore, OM treatment may be more suitable for foods with relatively low fat content.

### 3.3. Effects of OM Treatment on Protein Oxidation

#### 3.3.1. Free Amino Acids

Free amino acids are correlated to the nutritional value and flavor quality of meats [35]. In Table 3, complete data regarding the concentration of free amino acids in pork and fish samples treated by OM and CW are listed.

For pork samples, no significant difference was found in the 11 amino acids in the CW vs. OM comparison. However, significantly higher (*p* < 0.05) levels of Glycine (Gly), Alanine (Ala), and Proline (Pro) in the OM group were detected, which may originate from the higher proteolysis due to the change of protein secondary structure. As Jiang et al. [13] reported, ozone treatment led to a lower amount of α-helical and higher amount of β-sheet and random coil, which subsequently promoted protein unfolding and proteolysis [36]. However, significantly lower (*p* < 0.05) concentrations of Cysteine (Cys), Methionine (Met), Lysine (Lys), and Isoleucine (Ile) were determined in the OM group. Zhang et al. [37] also reported a significantly lower (*p* < 0.05) content of Cys, Met, and Lys in shrimp tropomyosin after 15 min of high-intensity ultrasound treatment, which induced free radicals in water, in comparison with the control group (without treatment). It may be explained by the fact that some amino acids, especially some sulfur amino acids such as Cys and Met, are susceptible to ROS [38], and some amino acids, especially Lys, are involved in the formation of more stable oxidized products by bonding covalently with some oxidized products [39]. Previous studies have explored the oxidation of amino acids caused by ozone, which indicated that many amino acids such as aromatic amino acids are highly reactive to ozone [40]. However, the contents of 10 free amino acids, such as aromatic amino acids including Phe and Tyr, were not significantly different (p > 0.05) between the two treatment groups.

For the fish samples, the content of Gly and Ala was significantly higher (*p* < 0.05) in the OM group. The reason for this result in fish samples may be similar to that of pork samples. Conversely, significantly lower (*p* < 0.05) contents of Serine (Ser) and Isoleucine (Ile) in the OM group were found. However, the levels of 13 amino acids did not show a significant difference in the CW vs. OM comparison.

Taken together, OM treatment may lead to higher proteolysis characterized as the higher levels of certain free amino acids and stronger pro-oxidative effects characterized as the lower levels of certain free amino acids compared with CW treatment. Therefore, the total free amino acids content showed no significant difference (*p* > 0.05) between the OM-treated samples and CW-treated samples.

#### 3.3.2. Oxidation Sites on Proteins

When food proteins are exposed to inactive molecular oxygen and ROS such as ozone, their interaction leads to oxidative modification situated at aliphatic, aromatic, heteroatom, or backbone sites [41,42]. To compare the degree of oxidation in proteins of the two treatments, HR-MS was used to detect the possible oxidative products and their exact positions.

- Types of oxidative modifications

**Figure 2:**
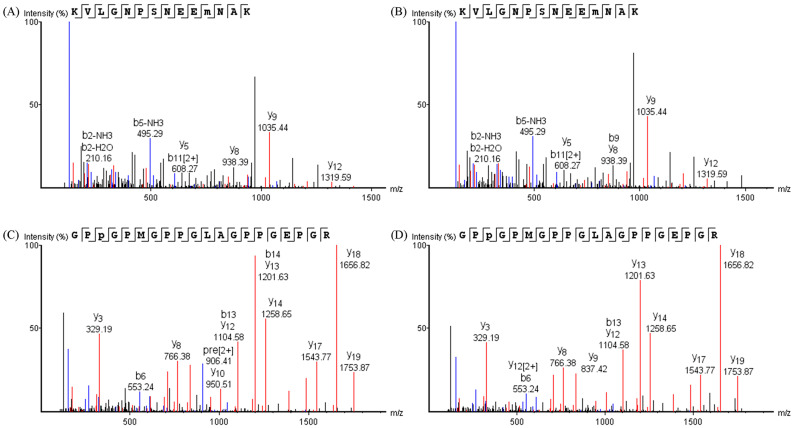
Representative examples of oxidatively modified peptides which were identified. MS/MS spectrum of the peptide KVLGNPSNEEM(O)NAK derived from pork samples treated with CW (**A**) and OM (**B**); MS/MS spectrum of the peptide GPP(O)GPMGPPGLAGPPGEPGR derived from fish samples treated with CW (**C**) and OM (**D**). CW: conventional water-washing; OM: ozone-microbubbles-washing.

B and y ion analysis is performed to detect protein oxidation sites and oxidation modification types. An example can be seen in Figure 2, where the MS/MS spectrum of the peptide KVLGNPSNEEM(O)NAK derived from MLC1f protein of pork samples and the peptide GPP(O)GPMGPPGLAGPPGEPGR from collagen type I alpha 1 protein of fish samples are shown, indicating that the same oxidative modification occurs in the CW and OM groups. Specifically, peptides of pork protein with Met monooxidation were found in the two treatments (Figure 2A,B), while peptides of fish protein with Pro monooxidation were found in the two treatments (Figure 2C,D).

**Figure 3:**
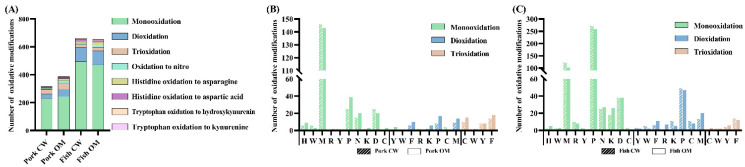
The number of oxidative modifications among all samples (**A**); oxidation sites and the number of monooxidation, dioxidation, and trioxidation modifications in pork sample (**B**); and fish sample (**C**). CW: conventional water-washing; OM: ozone-microbubbles-washing. M: Methionine; D: Aspartic Acid; P: Proline; N: Asparagine; Y: Tyrosine; K: Lysine; C: Cysteine; H: Histidine; W: Tryptophan; R: Arginine; F: Phenylalanine.

As shown in Figure 3A, the same eight oxidative modification types were detected in pork and fish samples with the CW and OM treatments. The numbers of oxidatively modified peptides detected in pork with CW and OM treatment are 232 and 277 (Tables S1 and S2), respectively. The numbers of oxidatively modified peptides detected in fish samples with CW and OM treatment are 505 and 489 (Tables S3 and S4), respectively. It shows that OM treatment has a slightly greater impact on the oxidation of pork protein, and the effects of OM treatment on the oxidation of fish protein is the same as that of CW treatment. In addition, the oxidative modifications of monooxidation, dioxidation, and trioxidation ranked as the top three oxidative modifications in both pork and fish samples with the two treatments. The modifications of Histidine oxidation to Aspartic Acid (Asp) and Asparagine (Asn) and oxidation to nitro also frequently occurred in various samples.

For pork samples (Figure 3B, Tables S1 and S2), the former three types of oxidative modification, as a whole, account for 93% and 87% of the entire modified peptides in the CW and OM groups, respectively. Compared with the two treatments, CW was more likely to promote the monooxidation of Met, Asp, Tyr, and Trp; OM was more likely to promote the monooxidation of Pro, Asn, His, Lys, Arg, and Cys, and the number of dioxidation and trioxidation in the OM group was slightly higher than that in the CW group on each modification site. In both CW and OM groups, most sites underwent monooxidation rather than dioxidation and trioxidation. Met, Pro, and Asp were the primary amino acid residues most prone to oxidation. Moreover, higher oxidation levels such as dioxidation and trioxidation occurred mainly in Phe and Tyr. This result was consistent with the previous study [43], which reported that dioxidation was prone to occur in aromatic amino acids. The regularity of the oxidative modification sites was also found in fish samples (Figure 3C, Tables S3 and S4), although the number of modifications of individual amino acids residues such as Pro varied.

- Types and properties of oxidatively modified proteins of pork samples

**Figure 4:**
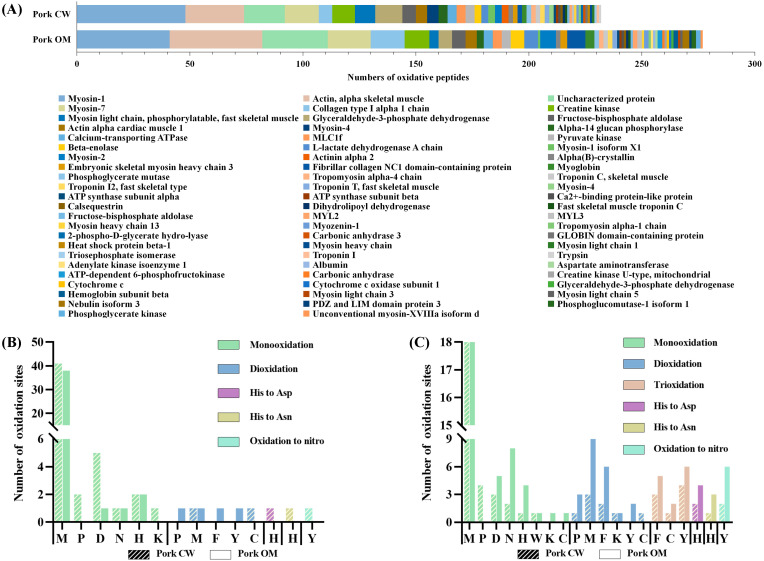
The number of oxidative peptides and the protein which they derived from (**A**); the type and the number of oxidative modifications in myosin-1 (**B**); the type and the number of oxidative modifications in actin, alpha skeletal muscle (**C**); CW: conventional water-washing; OM: ozone-microbubbles-washing. M: Methionine; D: Aspartic acid; P: Proline; N: Asparagine; Y: Tyrosine; K: Lysine; C: Cysteine; H: Histidine; W: Tryptophan; F: Phenylalanine. The protein name was according to the Uniprot database.

A total of 51 and 56 species of proteins were oxidized in the CW and OM groups, respectively. The detected peptides with oxidative modification were mainly derived from myosin, actin, and collagen in the two treatments (Figure 4A).

The largest number of peptides with oxidative modification were derived from different myosin family members. Myosin was prone to oxidize compared with actin, which was mainly due to the different structures [44]. Take myosin-1 as an example. With the highest proportion of oxidative modification, the main oxidative types were monooxidation and dioxidation in both treatments. In addition, modifications of Histidine oxidation to Aspartic Acid and Asparagine and oxidation to nitro were found in the CW group (Figure 4B). The numbers of total oxidative modifications in peptides of the CW group were similar but slightly higher than that of the OM group. In both CW and OM treatment groups, Met was the most vulnerable amino acid residue to oxidation because of the high activity of sulfur atoms [43]. In addition, Pro was more prone to dioxidation in the OM group, and monooxidation in the CW group. As is listed in Tables S1 and S2, for some sequence of peptides, such as EDQVFPMNPPKFDK, monooxidation occurred mainly in the CW group (EDQVFP(+15.99)MN(+15.99)PPKFDK, EDQVFP(+15.99)M(+15.99)NPPK), while dioxidation occurred in the OM group (EDQVFP(+31.99) MNPPKFDK). Since different types of oxidations occurred in similar sequences, the results of oxidative modification in both proteins were similar. From this aspect, OM treatment did not cause more severe oxidation.

For actin, take actin, alpha skeletal muscle as an example. Six types of oxidative modification were detected in both CW and OM groups (Figure 4C). Monooxidation, deoxidation, and trioxidation were the main types of oxidative modification, although the sensitivity of different amino acids residues varied in the CW vs. OM comparison. In addition, Met was the dominant site in CW and OM groups with the modification of monooxidation and dioxidation [45].

- Types and properties of oxidatively modified proteins of fish samples

**Figure 5:**
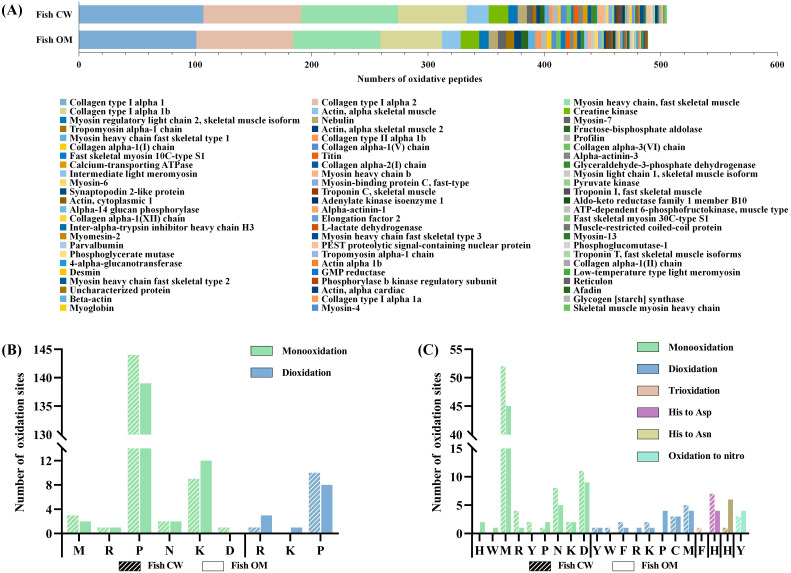
The number of oxidative peptides and the protein which they derived from (**A**); the type and the number of oxidative modifications in collagen type I alpha 2 (**B**); the type and the number of oxidative modifications in myosin, taking myosin heavy chain, fast skeletal muscle (**C**); CW: conventional water-washing; OM: ozone-microbubbles-washing. M: Methionine; D: Aspartic Acid; P: Proline; N: Asparagine; Y: Tyrosine; K: Lysine; C: Cysteine; H: Histidine; W: Tryptophan; R: Arginine; F: Phenylalanine. The protein name was according to the Uniprot database.

As for the fish sample, 61 and 64 species of proteins were oxidized in CW and OM groups, respectively, and the detected peptides with modification were mainly derived from myosin and collagen (Figure 5A).

As an essential structural protein in fish, mainly distributed in fish skin and bone [46], collagen contains more modifications than its counterpart proteins. Take collagen type I alpha 2 as an example. Both monooxidation and dioxidation were detected in the two treatments. The main modification sites in both treatment groups were Pro and Lys with monooxidation (Figure 5B). For these amino acid radicals, the mass shift of oxidation and hydroxylation was similar. Since hydroxylation was the critical type of modification in collagen, which occurred mainly at the Pro and Lys sites [47], it suggested that the main modification of Pro and Lys here was hydroxylation, which benefited collagen stability. In addition, the number of oxidative modifications in amino acid residues detected was roughly the same in the CW vs. OM comparison. For myosin, take myosin heavy chain, fast skeletal muscle as an example. Six types of oxidative modification were detected in both CW and OM groups, where the monooxidation of Met and Asp was prone to occur (Figure 5C). The oxidative modification of myosin of fish samples was similar to that of pork samples.

In summary, the oxidative modification of protein occurred in CW treatment; however, OM treatment did not massively aggravate the oxidation compared with CW treatment. The effects of the two treatments on protein oxidation was similar; however, the sensitivity of the modification sites of different proteins with different washing treatments varied, resulting in the various number of modifications of individual amino acid residues.

## 4. Conclusions

Ozone-microbubble-washing treatment performed by domestic ozone equipment did not cause damage to the integrity of muscle cells from both pork and fish samples, compared with the conventional water-washing treatment. The TBARS values were not detected in both treatment groups and were considerably below the generally acceptable threshold level (1 mg MDA/kg). Significantly lower contents of GO and MGO were found in OM-treated pork, while a higher content of MGO in OM-treated fish compared with CW-treated samples was found. In addition, the concentrations of 7 free amino acids (out of 17 measured) of pork and 4 free amino acids (out of 17 measured) of fish samples were significantly different in the CW vs. OM comparison, while the total free amino acid contents of both pork and fish samples with the two treatments did not differ. The oxidative modification of protein was similar in the two treatments, although the modification of individual amino acid residues varied. This study contributes to existing knowledge of the application of ozone by evaluating two species of muscle foods treated by ozone-microbubble-washing with domestic ozone equipment, which can give insight into the choice of domestic food washing treatments.

## Figures and Tables

**Table 1:** The identified oxidative modifications of amino acid residues under the impact of two washing treatments.

Modification	Amino Acid Residue	Position	Formula	Mass Shift (Da)
Monooxidation	HWMRYPNKDC	any	O	15.99
Dioxidation	YWFRKPCM	any	O(2)	31.99
Trioxidation	CWYF	any	O(3)	47.98
Tryptophan oxidation to kynurenine	W	any	C(−1)O	3.99
Oxidation to nitro	YW	any	H(−1)NO(2)	44.99
Tryptophan oxidation to hydroxykynurenin	W	any	C(−1)O(2)	19.99
Histidine oxidation to Aspartic Acid	H	any	H(−1)C(−2)N(−1)O	−22.03
Histidine oxidation to Asparagine	H	any	H(−2)C(−2)N(−2)O(2)	−23.02

**Table 2:** The amount of thiobarbituric acid reactive substances (TBARS) values (mg MDA/kg sample), GO (µg/kg sample), and MGO (µg/kg sample) in pork and fish samples under conventional water-washing (CW) treatment and ozone-microbubble-washing (OM) treatment.

Compounds	Pork		Fish	
	CW	OM	CW	OM
TBRAS value	ND	ND	ND	ND
GO	23.21 ± 1.90	17.13 ± 2.73 *	27.60 ± 0.37	26.93 ± 0.55
MGO	72.23 ± 4.77	55.23 ± 7.58 *	100.55 ± 4.18	118.93 ± 3.99 *

ND indicates that TBARS value was not detected (level of detection was 0.05 mg MDA/kg). MDA: malondialdehyde; GO: glyoxal; MGO: methylglyoxal. For each species of muscle foods, * indicates that the values in the same row are significantly different (*p* < 0.05) between the two different washing treatment groups.

**Table 3:** Free amino acid composition (µg/g) in pork and fish samples under conventional water-washing (CW) treatment and ozone-microbubble-washing (OM) treatment.

Free Amino Acid	Pork		Fish	
	CW	OM	CW	OM
Asp	13.28 ± 2.26	12.91 ± 0.15	10.44 ± 0.10	11.01 ± 0.94
Ser	39.58 ± 1.47	37.34 ± 0.70	34.73 ± 1.58	28.67 ± 1.61 *
Glu	50.77 ± 4.40	56.11 ± 1.43	20.98 ± 0.96	24.61 ± 3.04
Gly	46.84 ± 1.10	53.01 ± 0.80 *	484.23 ± 16.79	531.03 ± 9.21 *
His	20.12 ± 1.24	19.17 ± 0.35	1121.46 ± 22.93	1145.86 ± 24.65
Arg	36.52 ± 6.61	42.91 ± 6.78	102.73 ± 6.02	116.61 ± 9.07
Thr	1735.14 ± 76.58	1736.48 ± 5.25	ND	ND
Ala	307.21 ± 9.41	354.44 ± 2.11 *	176.12 ± 13.00	291.46 ± 31.39 *
Pro	24.33 ± 3.17	66.95 ± 1.26 *	138.46 ± 6.94	125.32 ± 13.42
Cys	30.28 ± 4.78	19.36 ± 0.39 *	ND	ND
Tyr	23.12 ± 3.36	18.81 ± 0.44	4.45 ± 1.44	4.88 ± 0.76
Val	27.74 ± 6.66	25.20 ± 5.60	21.51 ± 2.13	22.19 ± 2.47
Met	12.38 ± 1.19	7.12 ± 0.20 *	8.74 ± 1.16	8.51 ± 1.07
Lys	23.89 ± 2.39	13.76 ± 0.15 *	139.4 ± 14.09	146.34 ± 17.03
Ile	17.38 ± 2.04	11.58 ± 0.29 *	29.81 ± 2.49	23.75 ± 2.34 *
Leu	30.55 ± 2.27	27.53 ± 0.48	44.29 ± 3.08	44.74 ± 3.53
Phe	14.67 ± 1.32	12.64 ± 0.50	15.39 ± 1.16	13.49 ± 0.57
Total FAA	2453.78 ± 98.51	2515.33 ± 7.25	2352.74 ± 87.92	2538.46 ± 108.78

ND indicates that the compound was not detected. For each species of muscle foods, * indicates that the values in the same row differ significantly (*p* < 0.05) between the two different washing treatment groups.

## Data Availability

Data presented in this study are available on request.

## Supplementary Materials

The following are available online at https://www.mdpi.com/article/10.3390/foods11070903/s1, Table S1: Detailed information for the oxidatively modified peptides identified in pork samples treated by CW; Table S2: Detailed information for the oxidatively modified peptides identified in pork samples treated by OM; Table S3: Detailed information for the oxidatively modified peptides identified in fish samples treated by CW; Table S4: Detailed information for the oxidatively modified peptides identified in pork samples treated by OM. Information including sequence, PTMs, score (−10lgP), length, protein names, and accession no. are provided in the tables.
